# Spatiotemporal Dynamics of Affective and Semantic Valence Among Women

**DOI:** 10.3389/fnhum.2021.602192

**Published:** 2021-07-13

**Authors:** Luchun Wang, Xiying Li, Zhongling Pi, Shuoqi Xiang, Xuemei Yao, Senqing Qi

**Affiliations:** ^1^Key Laboratory of Modern Teaching Technology, Ministry of Education, Shaanxi Normal University, Xi'an, China; ^2^School of Foreign Languages, Xi'an University of Finance and Economics, Xi'an, China

**Keywords:** emotion, affective valence, semantic valence, habituation, LPP

## Abstract

As an important dimension of emotional assessment, valence can refer to affective valence reflecting an emotional response, or semantic valence reflecting knowledge about the nature of a stimulus. A previous study has used repeated exposure to separate these two similar cognitive processes. Here, for the first time, we compared the spatiotemporal dynamics of the affective and semantic modes of valence by combining event-related potentials with repeated exposure. Forty-seven female participants were assigned to the feeling-focused and semantic-focused groups and thereafter repeatedly viewed the pictures selected for the study. Self-report behavioral results showed that post-test scores were significantly lower than pre-test scores in the feeling-focused group, while the differences between the two tests were not significant in the semantic-focused group. At the neural level, N2 amplitudes decreased and early late positive potential amplitudes increased in both groups, suggesting that the participants perceived the repeated pictures more fluently and retrieved the traces of the stimulus spontaneously regardless of the valence they judged. However, the late positive potential amplitudes in anterior areas and the activity of the middle frontal gyrus were attenuated in the feeling-focused group; however, this component in posterior areas and the activity of the precentral gyrus were increased in the semantic-focused group. Therefore, the processes of affective and semantic valence are similar in the early stages of image perception and retrieval, while in the later stage of valence judgment, these processes show different brain activation patterns. The results provide electrophysiological evidence for the differences in psychological processes when judging the two modes of valence.

## Introduction

Valence, a primary dimension of emotion, is commonly reflected in self-reports or other measurements in emotional research (Lang et al., 1993). When talking about the valence of emotional stimuli, people use labels such as “happy/sad” and “positive/negative” to describe different affective states. Among them, “I feel happy when I see a family photo” tends to describe the valence of inner emotional feelings subjectively, while “This is a happy family photo” tends to describe the valence of events or objects objectively. Emotional feelings and semantic knowledge are different patterns of determining valence. Based on these differences, Itkes et al. (2017) have posited that there are two modes of valence: affective (the valence of emotional response) and semantic (the knowledge about the positivity or negativity of events or objects).

Although these two modes of valence are defined differently, they may coexist in the judgment process, so it is not easy to separate them. An assessment of an event or object is part of the objective processes of evaluation, attitudes, etc. These assessment processes sometimes occur accompanied by the activation of the peripheral neurophysiology of emotional sensations (Russell, 2017). Similarly, Itkes et al. (2019) found that when presenting emotional pictures, self-report scores about mixed evaluation as both pleasant and unpleasant involved a higher proportion of semantic knowledge component and a lower proportion of feelings component. In other words, self-reported scores contained both semantic and feeling components. Furthermore, Itkes and Kron (2019) discussed five kinds of typical examples of the confusion of the two modes of valence in empirical research. Many studies did not clearly distinguish the two modes of valence in their instructions, which led to inconsistent results (Lazarus and Smith, 1988). Therefore, dissociating the affective and semantic representations of valence and elucidating the distinction of psychological processes between them are essential for valence-related studies.

Some theoretical models showed a similar distinction between “emotional feelings” and “cognition about emotions.” For example, the accessibility model posited that emotional self-reports under short vs. long time frames were based on different sources of knowledge. People retrieve episodic knowledge based on their feelings (experiential) over short time frames but retrieve semantic (non-experiential) knowledge based on the beliefs of the event over long time frames (Robinson and Clore, 2002). In addition, Russell (2017) argued that an affective reaction includes not only the subjective feeling and its physiological correlates (core affect) but also the objective judgment of the event or stimuli (affective quality). These views are compatible with affective valence and semantic valence in this study. Thus, these variations of the similar distinction theoretically imply different cognition processes between affective and semantic valence. Empirical methods should be used to show the neurophysiological mechanisms between the two modes of valence.

To separate affective valence and semantic valence effectively, Itkes et al. (2017) have used repeated exposure in the habituation paradigm to demonstrate their differences. In their study, the intensity of affective valence (emotional feelings) decreased after repeated viewing, as reflected in self-reports and peripheral physiological indicators, but the intensity of semantic valence did not. This method has indicated that affective valence reflects an inner emotional response, which is susceptible to the influence of habituation. Semantic valence reflects relatively stable stored knowledge. Further correlational analyses showed that the relationship between affective- and semantic-related measures was highly intercorrelated during the first exposure of an individual. However, the correlation of the slopes between these two measures, that is, the score changes between first and last exposure to the stimuli (first minus last), was lower. Thus, the two modes of valence are highly correlated but separable, probably because the two processes have both similarities and differences.

The study of Itkes et al. (2017) has also indicated that repeated exposure is an effective way to separate the two modes of valence. However, they have not directly uncovered the neural mechanisms underlying these two modes of valence. Therefore, this study would combine repeated exposure with event-related potentials (ERPs). ERP components offer unique insight into the brain resources allocated to the processes of affective pictures caused by repeated exposure (Ferrari et al., 2017). They can provide direct and functionally separable evidence of the differences in the neural mechanisms of judging affective and semantic valence.

In this study, the participants were divided into a feeling-focused group and a semantic-focused group. They judged the valence for pictures after reading instructions about affective and semantic valence, respectively. According to the peripheral physiological and behavioral reactions in the study of Itkes et al. (2017), we predicted that affective and semantic valence judgements differed before and after repeated viewing. These differences were manifested evidently in the changes in both valence scores and ERP components. The following paragraphs specify the functional significance of ERP components during the repetitive processing of the pictures.

Prior ERP studies have investigated the changes in the early and late components before and after repetitive viewing. First, in the early stage of approximately 200–300 ms, repetitions can reduce the amplitudes of anterior N2 both for emotional and neutral pictures, indicating that N2 amplitudes were modulated by novel stimuli but not valence of stimuli (Ferrari et al., 2010). This reduction is explained as increased perceptual fluency, which means that if the stimulus has been presented before, the prior occurrence of this stimulus builds a short-term memory template that promotes the perception of it (Ferrari et al., 2015, 2017).

Second, suppression or enhancement of late positive potential (LPP) amplitudes in different time windows and locations caused by repetition might reflect different psychological processes, such as memory, habituation, or trace retrieval (Ferrari et al., 2017). In particular, distributed repetition, in which there was a temporal distance between the presentations of the same stimuli, increased the LPP amplitudes during the 500–800 ms period (Curran and Doyle, 2011; Ferrari et al., 2015). This LPP enhancement was similar to the old-new effect of the recognition task in timing and topography. In the previous recognition task, if participants were asked to identify whether the stimulus was previously presented or new, the old stimulus (repeated viewing) elicited an LPP amplitude larger than the new stimulus (first viewing). Further research showed that even without the explicit requirement of recognition, old stimuli elicited enhanced LPP amplitudes relative to the new item. This was explained as reflecting the spontaneous retrieval of previously encountered items after ~500–800 ms (Weymar et al., 2013b, 2014).

In addition, studies have proposed that emotional responses are sensitive to habituation. They can change under the influence of repetitions, while semantic knowledge is category-specific and not easy to change because of its stable conceptual properties (Thompson-Schill, 1999; Beedie et al., 2005). Several electrophysiological studies have shown that as the number of repetitions increases, LPP amplitudes in the relatively late stage and peripheral reactions related to emotional feelings decline (Codispoti et al., 2006, 2016). However, during the repeated viewing process, when subjects continued extracting semantic information from pictures, they imagined a complete background story based on their existing knowledge. This process represented continuous semantic elaboration (Bauer and Jackson, 2015). A functional magnetic resonance imaging (fMRI) study has shown that the semantic elaboration process enhances the activation of related brain regions associated with semantic processing (Kaneda et al., 2017). Therefore, the judgment of affective and semantic valence may lead to distinct LPP performances in the late stage. Based on these different cognition processes, the time window of the LPPs in this study was divided into early and late stages. Also, when affective images were processed, the LPPs may have distributed from posterior sites to all scalp regions (Hajcak and Olvet, 2008; Gao et al., 2010). Standardized low-resolution electromagnetic tomography (sLORETA) can provide information regarding the neural origin during processing. Thus, we calculated the sources of the current density of the LPP components for the different modes of valence.

Based on the above ERP evidence for processing affective pictures *via* repeated exposure, we expected different spatiotemporal dynamics of the two modes of valence. In conjunction with this, we first expected a significant decrease in N2 component amplitudes for both groups, regardless of the kinds of valence that the participants judged. Second, distributed repetition elicited the enhancement of the LPPs in the early time window for both groups, representing the recognition of repeated old stimuli. In the first two hypotheses, we proposed that the early cognition processes of the two modes of valence would be similar. However, based on the different characteristics and judgment criteria of the affective and semantic valence, LPP amplitudes in the late stage for the two groups would be different. Third, the emotional feelings of the feeling-focused group were reduced when the participants saw the pictures because of habituation, as reflected in the LPP amplitudes and self-reports. Finally, the semantic-focused group extracted the valence information of the pictures from semantic knowledge, resulting in enhanced LPP amplitudes and similar self-reports after repeated viewing.

In conclusion, we assumed that the early processing of the two modes of valence was similar but that the late processing was different. The application of ERPs in conjunction with sLORETA may help improve the present understanding of the different neural activations of the two modes of valence from the perspective of time course and scalp distribution. Therefore, behavioral performance and brain function would be connected.

## Method

### Participants

Forty-seven female participants (age: M = 19.23 years, SD = 0.79) volunteered in the study. Only females were chosen to control any gender differences in affective habituation (Andreano et al., 2014). Additionally, as most Shaanxi Normal University classes were dominated by female students, there was an insufficient number of male participants in the sample recruitment. All the participants were right-handed and had a normal or corrected-to-normal vision. They reported no history of psychiatric or neurological disease and gave informed consent prior to participating. The local ethics committee of the Shaanxi Normal University approved this study.

### Material

#### Stimuli

We selected 80 images from the International Affective Picture System (IAPS) (Lang et al., 2008) and 28 from the internet. All the pictures were properly formatted with Photoshop 7.0: the picture size was 1,024 × 768 with a resolution of 72 DPI. A total of 108 pictures were assessed by another group of 21 female participants (age: M = 18.71 years, SD = 0.85). Ratings were obtained using a 9-point scale in terms of valence and arousal: valence (1 = very unpleasant, 9 = very pleasant), arousal (1 = calm, 9 = excited). Repeated-measures analyses of variance (ANOVA) were conducted to examine the valence and arousal of the categories. Finally, a total of 36 pictures from the IAPS^1^ and the internet were matched in the formal research (12 pictures for each stimulus type). The main effects of valence and arousal were significant [valence: *F*_(2, 40)_ = 250.71, *p* < 0.001, η_*p*_^2^ = 0.93; arousal: *F*_(2, 40)_ = 76.44, *p* < 0.001, η_*p*_^2^ = 0.79]. Pairwise comparisons showed that emotional pictures differed significantly from the neutral pictures in valence and arousal for both groups (*p*s < 0.001). Positive pictures showed higher scores than negative pictures in valence (*p* < 0.001) but no significant difference in arousal (*p* = 1.00). Table 1 presents the means and standard deviations of valence scores and arousal scores of the three types of pictures.

**Table 1:** Means (standard deviations) of valence scores and arousal scores of the three types of pictures.

	**Positive**	**Negative**	**Neutral**
Valence	7.18 (0.70)	2.55 (0.74)	5.00 (0.31)
Arousal	5.87 (1.47)	6.09 (1.18)	3.37 (1.08)

The positive pictures depicted appetizing food, lovely animals, pleasant family scenes, pleasant nature, and romantic heterosexual couples. The negative pictures included environmental contamination, injured human beings, terrible disaster, and suffering animals. The neutral pictures showed household objects, common buildings, and neutral human activities.

#### Task Instructions and Rating Scales

The participants were randomly assigned into one of the two instruction groups: the feeling-focused and semantic-focused groups. We used the study of Itkes et al. (2017) for reference to develop different instructions. At the beginning of the experiment, the participants read the instructions of affective valence and semantic valence. Differences between them were emphasized. For example, when looking at a picture of an amusement park, someone may not have a strong and happy feeling (affective valence). However, people know that going to the amusement park is a positive event (semantic valence). For feeling-focused group instructions, the participants were asked to experience their inner feelings and evaluate the intensity of affective valence (e.g., happiness and unhappiness). For semantic-focused group instructions, the participants were asked to retrieve the semantic knowledge related to events or objects in the pictures from daily experiences and evaluate the intensity of their semantic valence (e.g., positivity and negativity). To ensure that the participants understood the instructions correctly, we provided them with three non-experimental pictures to rate after reading the instructions. Each participant told us about her rating criterion. If the rating criterion was consistent with the instructions, the participant could start the formal experiment.

According to previous studies (Kron et al., 2013, 2015; Itkes et al., 2017), the unipolar valence model supports that happiness and unhappiness are two independent dimensions. Therefore, three separate unipolar scales of valence rating from 0 (none) to 8 (high) were used in the feeling-focused group: the overall emotional intensity scale (rating the intensity of “general” emotional feelings whether positive or negative), pleasant scale (rating feelings such as happiness, excitement, and pleasure), and unpleasant scale (rating feelings such as sadness and unhappiness). The first scale was used to remind the participants that this was an emotional detection task when experiencing low emotional feelings so as to avoid reporting semantic knowledge. Two rating scales in the semantic-focused group were used: positive and negative scales. These two scales were the same as the pleasant and unpleasant scales in the feeling-focused group. However, the intensity was based on evaluating how positive or negative the picture content was but not the feelings.

### Procedure

After entering the laboratory, both groups of participants learned the requirements of the instruction carefully. Then, they were connected to electroencephalogram (EEG) electrodes and randomly assigned to one of the two instruction groups. The whole experiment consisted of three phases: the first and last phases were rating stages, and the second phase was a repeated stage. Three emotional scales and two semantic scales were used in the feeling-focused and semantic-focused groups, respectively. After ensuring that the participants understood the instructions, they began the first rating phase and rated the valence of each picture. Each trial started with a white fixation cross-presented on a black screen for 0.5 s. The fixation cross disappeared and then a picture was presented for 2.5 s. After the offset of each picture, unlimited time was allowed to rate each picture using three or two scales in terms of valence. There were 36 trials in the first rating phase, with each picture presented once.

After all the pictures were rated, the participants started the second phase consisting of twelve blocks. Each picture was presented for 2.5 s. The 36 pictures were repeated once randomly in each block, so each picture was repeated 12 times in this phase. The participants needed to view the pictures carefully without doing anything during this phase.

In the final phase, the procedures and instructions were the same as those in the first phase of each group. The different groups still had different instructions. Specifically, the feeling-focused group was required to rate the pictures based on the emotional experience at that moment, while the semantic group was required to rate the pictures based on the evaluation of the picture content at the moment.

### Electroencephalogram Recording

Electroencephalogram data were recorded at a sampling rate of 500 Hz from 64 scalp sites using Ag/AgCl electrodes with a Neuroscan recording system and referenced to the left mastoid, with a ground electrode in the medial frontal aspect (SynAmps2 amplifier, DC-100 Hz). The vertical electrooculogram (EOG) was recorded supra- and infraorbitally in the left eye; and the horizontal EOG signals were recorded from the left vs. the right orbital rim. For all of the electrodes, impedance was kept under 5 KΩ.

The EEG signals were evaluated in MATLAB using the EEGLAB (Delorme and Makeig, 2004) and ERPLAB toolboxes (Lopez-Calderon and Luck, 2014). After removing muscle artifacts or extreme offsets, all EEG data were re-referenced to the average of the left and right mastoids and filtered using Butterworth filters with half-power cutoffs at 0.1 and 30 Hz (roll-off = 12 dB/octave). The components associated with eye movements or eyeblink activities were removed by independent component analysis (ICA) (Jung et al., 2000). The remaining ICA-corrected EEGs were segmented into epochs. Baseline correction was performed by subtracting the mean of 200 ms prior to stimulus onset. Any epoch with EEG voltages exceeding the threshold of ±100 μV was excluded from the average. The percentage of trials excluded from averaging because of artifact detection was 10.30% for the feeling-focused group and 10.27% for the semantic-focused group.

### Data Processing

The ICA-corrected EEG data were segmented into epochs that began 200 ms before the onset of the picture and continued to 2,500 ms for analysis. ERPs in the pre-test were averaged by the first and second blocks, whereas ERPs in the post-test were averaged by the 13th and the 14th blocks. Based on previous studies (Codispoti et al., 2007; Folstein et al., 2008), repetition had different effects on the two components. The first component is the early frontal N2 component, which occurs ~200–300 ms after stimulus onset. This is a negative component that is reliably enhanced by novel visual stimuli (Ferrari et al., 2015). Thus, the N2 amplitude was averaged at five frontal–central hemispheric electrodes (FCZ, FC1, FC2, FC3, and FC4) in the time interval of 210–280 ms. The second component is the LPP, which reflects the processing of affective stimuli. The frontal LPP amplitudes were averaged by three electrodes (Fz, F1, and F2); central–parietal LPP amplitudes were quantified as the average activity collapsed across three electrodes (CP1, CPz, and CP2); and parietal–occipital LPP amplitudes were calculated by the averaged amplitudes across three electrodes (POZ, PO3, and PO4), each of which was quantified over two time windows: early LPP (400–1,000 ms) and LPP (1,000–2,500 ms).

On the basis of a previous study (Itkes et al., 2017), the effects of repetition were examined by the difference between the tests of the first presentation (pre-test) and the last presentation (post-test) of the same picture in behavioral data. For statistical analyses, average valence scores and mean amplitude of the N2 component were entered into three-way repeated-measures ANOVA with group (the feeling-focused group and the semantic-focused group) as a between-subject factor and with valence (positive, negative, and neutral) and test (pre-test and post-test) as within-subject factors. The resulting LPP amplitudes were entered into four-way repeated-measures ANOVA with region (frontal, central–parietal, and parietal–occipital), valence (positive, negative, and neutral), and test (pre-test and post-test) as within-subject factors and participant group (the feeling-focused and semantic-focused groups) as the between-subject factor for each time window. In addition to these analyses, we separated the groups and conducted additional two-way and three-way ANOVAs for N2 and LPP amplitudes, respectively, to assess the effects of repetition on emotional habituation and semantic processes. We pursued this method because valence and region may cover the nature of differences between the groups in this study. All the repeated-measures ANOVAs were Greenhouse–Geisser corrected; simple-simple effects analyses and *post-hoc* multiple comparisons were performed using Bonferroni corrections after significant interactions.

### Source Analyses (Standardized Low-Resolution Electromagnetic Tomography)

sLORETA has always been considered as an efficient functional localization method (Pascual-Marqui, 2002). In this study, it is helpful to explore the difference in source location of the LPP components between pre-test and post-test. sLORETA partitioned the intracerebral volume into 6,239 voxels. The transformation matrix was performed using the electrode coordinates generated by the 63 original electrodes (Jurcak et al., 2007). The averaged waveforms for all 1,350 time samples were converted into ASC values for the pre-test and post-test sessions for each subject. To determine the difference between the pre- and post-tests in a temporal and specific space, paired *t*-tests were computed for all the time samples in the feeling-focused group and the semantic-focused group. Average reference and 5,000 randomization tests based on the statistical non-parametric mapping (SnPM) method were performed (for details, see Nichols and Holmes, 2001). Each statistically significant voxel (*p* < 0.05) within the time range of the LPP was located. Finally, the regions with significant differences between pre-test and post-test sessions were plotted for each group. The areas with the largest differences in the Montreal Neurological Institute (MNI) brain template and Brodmann areas (BAs) are reported.

## Results

### Self-Report Measurement

**Figure 1:**
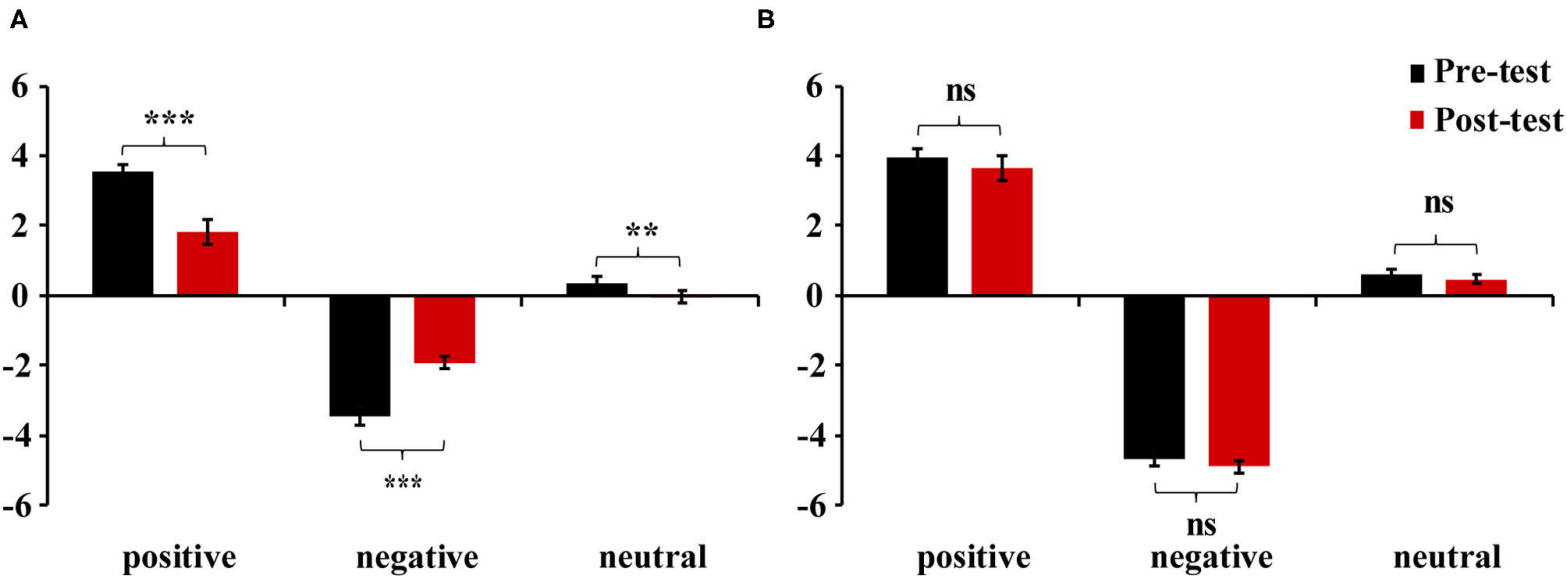
Mean valence scores of positive, negative, and neutral pictures in **(A)** feeling-focused group and **(B)** semantic-focused group. Error bars represent the standard errors of the means. **p < 0.01, ***p < 0.001, *ns* > 0.05.

We estimated bipolar valence scores as the final results for self-reports (Kron et al., 2015). Thus, Table 2 presents the two unipolar self-report scores of pleasure and displeasure, which are converted into a single bipolar valence score (positive minus negative) for the purpose of simplifying data analysis. The analysis of mixed variance was performed. As hypothesis 1 predicted, Group × Valence × Test interaction for valence ratings was significant, *F*_(2, 90)_ = 55.32, *p* < 0.01, η_*p*_^2^ = 0.55. Specifically, simple-simple effect analyses showed that the feeling-focused group had significantly higher rating scores in the pre-test than in the post-test among the three kinds of pictures (*p*s < 0.01). However, the semantic-focused group had similar scores between the two tests among the three kinds of pictures (*p*s > 0.05). No other main or other interaction effects were significant (*p*s > 0.05). Figure 1 shows that after repeated viewing, the self-report scores of the feeling-focused group decreased, while those of the semantic group did not change significantly.

**Table 2:** Means (standard deviations) of valence scores between pre-test and post-test for three valence categories in two groups.

**Measure**	**Group**	**Valence**	**Pre-test**	**Post-test**
Valence scores	Feeling-focused Group	Positive	3.53 (0.31)	1.81 (0.32)
		Negative	−3.46 (0.22)	−1.92 (0.21)
		Neutral	0.34 (0.20)	−0.05 (0.14)
	Semantic-focused Group	Positive	3.95 (0.29)	3.63 (0.30)
		Negative	−4.66 (0.21)	−4.90 (0.20)
		Neutral	0.62 (0.19)	0.46 (0.13)

### Event-Related Potential Data

#### N2 (210–280 ms)

**Figure 2:**
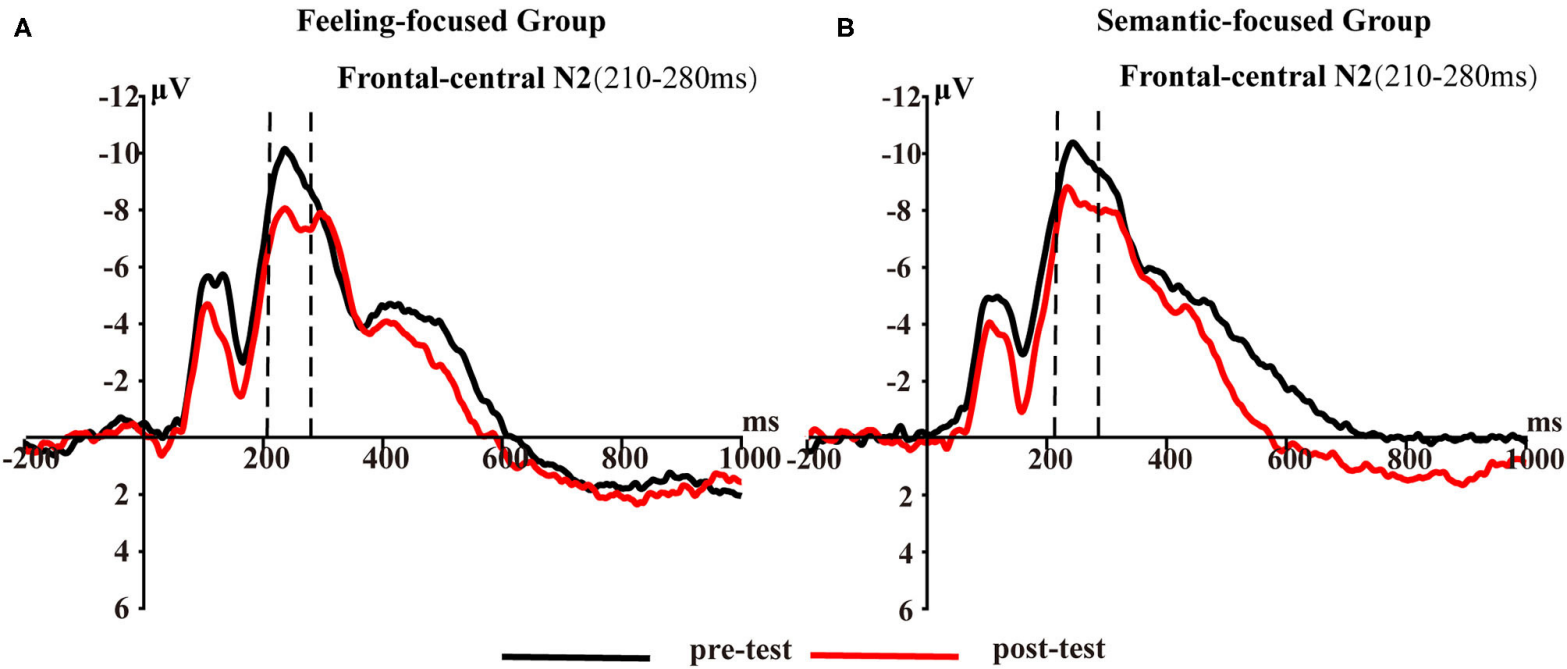
Frontal–central N2 averaged at five frontal–central sites (FCZ, FC1, FC2, FC3, and FC4) for **(A)** feeling-focused group and **(B)** semantic-focused group in pre- and post-tests for 210–280 ms.

Figure 2 presents frontal-central N2 waveforms elicited by pictures in the first and last viewing phases for each group. To test hypothesis 2, Group × Test repeated-measures ANOVA was conducted. The interaction was not significant [*F*_(1, 45)_ = 0.21, *p* = 0.65, η_*p*_^2^ = 0.005]. However, the main effect of the test was significant [*F*_(1, 45)_ = 17.65, *p* < 0.001, η_*p*_^2^ = 0.28]. A *post-hoc* test showed that the N2 amplitude in the pre-test (*M* = −9.4, *SD* = 1.3) was higher than that in the post-test (*M* = −7.56, *SD* = 1.11) in the feeling-focused group. These results were similar to those of the semantic-focused group. The N2 amplitude in the pre-test (*M* = −9.65, *SD* = 1.22) was higher than that in the post-test (*M* = −8.17, *SD* = 1.04) in the semantic-focused group. These results indicated that repeated viewing of pictures may lead to a significant decrease in N2 amplitude regardless of the valence of pictures and instruction requirements.

#### Early Late Positive Potential (400–1,000 ms)

**Figure 3:**
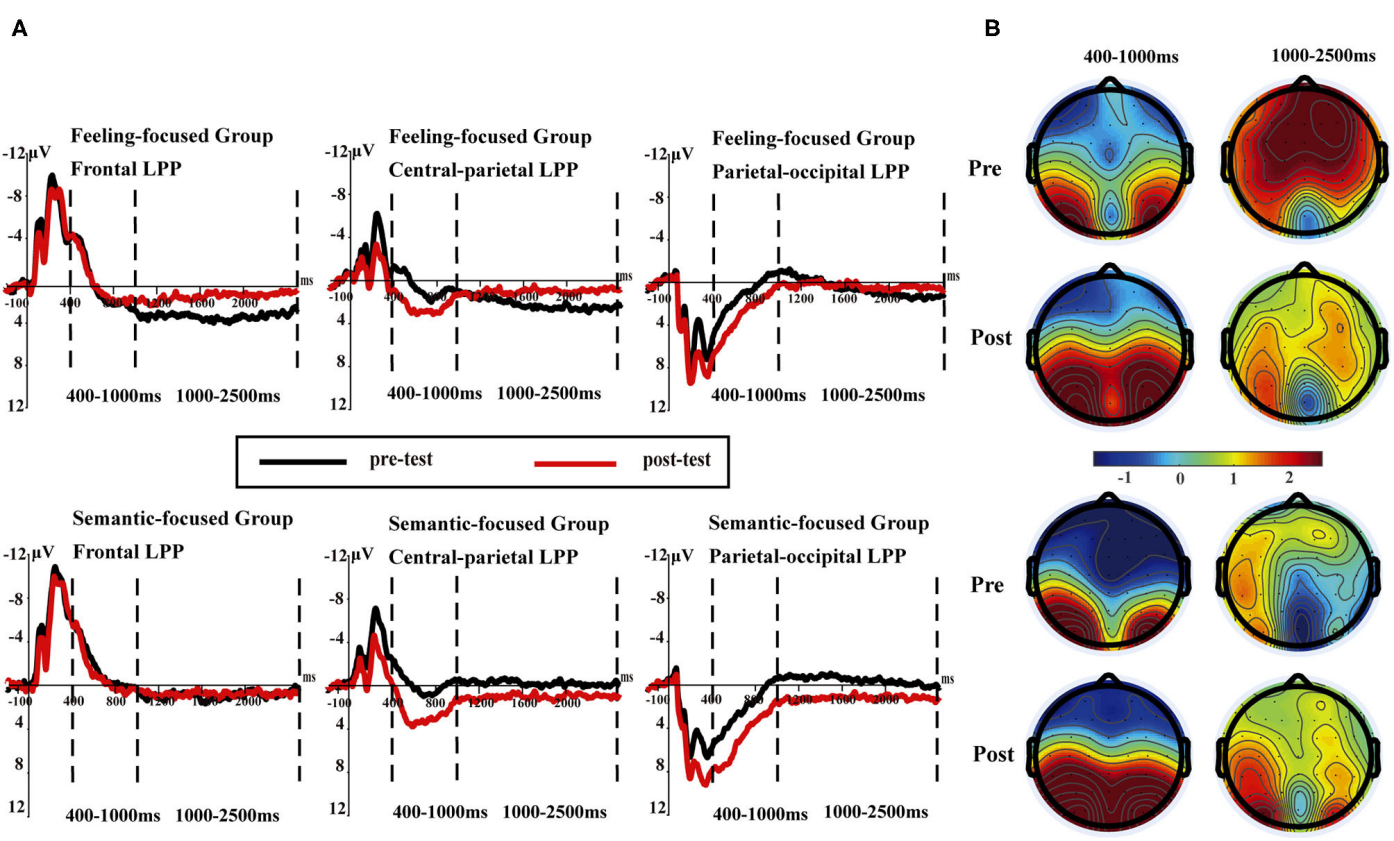
**(A)** Late positive potentials averaged at three sites (F1, FZ, F2; CP1, CPz, CP2; and PO3, POz, PO4) in pre- and post-tests for the feeling-focused (upper panel) and semantic-focused groups (lower panel) in 400–1,000 and 1,000–2,500 ms, respectively. **(B)** The scalp distribution of pre- and post-tests in 400–1,000 and 1,000–2,500 ms for both groups.

Figure 3 presents the early LPP waveforms of the two groups at different electrode sites at 400–1,000 ms. A mixed design repeated-measures ANOVA examining early LPP amplitudes revealed that the interaction of four variables was not significant [*F*_(4, 180)_ = 0.23, *p* = 0.92, η_*p*_^2^ = 0.01]. To test hypotheses 3 and 4, we separated the two groups of data and conducted Valence × Test × Region analyses for each group.

In the feeling-focused group, the main effects of the region [*F*(2, 42) = 5.93, *p* = 0.005, η_*p*_^2^ = 0.22], valence [*F*_(2, 42)_ = 21.6, *p* < 0.05, η_*p*_^2^ = 0.51], and test [*F*_(1, 21)_ = 4.78, *p* = 0.04, η_*p*_^2^ = 0.19] were significant. Importantly, the interaction between test and region was significant [*F*_(2, 42)_ = 11.86, *p* < 0.05, η_*p*_^2^ = 0.36], and other interactions were not significant (*p*s > 0.05). Further analyses showed that the frontal LPP amplitude elicited in the pre-test was similar to that in the post-test (*p* = 0.83). However, the central-parietal and parietal-occipital LPP amplitudes in the post-test were higher than those in the pre-test (*p* = 0.007, *p* = 0.001, respectively).

Consistent with these results, in the semantic focused group, the significant main effects of region [*F*_(2, 48)_ = 38.19, *p* < 0.001, η_*p*_^2^ = 0.61], valence [*F*_(2, 48)_ = 11.89, *p* < 0.001, η_*p*_^2^ = 0.33], and test [*F*_(1, 24)_ = 12.62, *p* = 0.002, η_*p*_^2^ = 0.35] were also found. Importantly, the interaction between region and test was significant [*F*_(2, 48)_ = 14.16, *p* < 0.001, η_*p*_^2^ = 0.37]. Simple effect analyses showed that the central-parietal and parietal–occipital LPP amplitudes in the post-test were higher than those in the pre-test (*p*s < 0.001) (see Table 3). The difference in the frontal LPP in the pre-test was not significant compared with that in the post-test (*p* = 0.56).

**Table 3:** Means (standard deviations) for early LPP (400–1000 ms) and late LPP (1000–2500 ms) between pre-tests and post-tests at each region in two groups.

**Measures**	**Group**	**Region**	**Pre-test**	**Post-test**
400–1,000 ms early LPP	Feeling-focused group	Frontal	0.27 (0.72)	0.41 (0.74)
		Central-parietal	0.61 0.61)	2.35 (0.52)
		Parietal-occipital	1.17 (0.71)	2.98 (0.59)
	Semantic-focused group	Frontal	−1.57 (0.96)	−1.13 (0.67)
		Central-parietal	−0.13 (0.84)	2.55 (0.62)
		Parietal-occipital	2.28 (0.69)	4.80 (0.55)
1,000–2,500 ms late LPP	Feeling-focused group	Frontal	2.95 (0.49)	0.98 (0.60)
		Central-parietal	2.19 (0.46)	1.02 (0.32)
		Parietal-occipital	0.52 (0.45)	0.35 (0.44)
	Semantic-focused group	Frontal	0.87 (0.50)	0.74 (0.42)
		Central-parietal	−0.24 (0.48)	1.02 (0.42)
		Parietal-occipital	−0.45 (0.49)	1.21 (0.46)

The results of early LPP amplitudes in both groups showed similar dynamic changes. Early LPP amplitudes in the post-test were more positive than those in the pre-test in the central–parietal and parietal–occipital areas, which represented similar cognition processes for the two groups.

#### Late Positive Potential (1,000–2,500 ms)

During the time window of 1,000–2,500 ms, repeated-measures ANOVA indicated that the Group × Region × Valence × Test interaction was not significant [*F*_(4, 180)_ = 0.41, *p* = 0.81, η_*p*_^2^ = 0.009]. However, to test hypotheses 3 and 4, we separated the two groups and performed the calculation separately. Figure 3 presents the LPP waveforms of the two groups at different electrode sites at 1,000–2,500 ms.

In the feeling-focused group, the main effects of region [*F*_(2, 42)_ = 5.53, *p* = 0.007, η_*p*_^2^ = 0.21], valence [*F*_(2, 42)_ = 4.07, *p* = 0.02, η_*p*_^2^ = 0.16], and test [*F*_(1, 21)_ = 13.29, *p* = 0.002, η_*p*_^2^ = 0.39] were significant. The Region × Test interaction [*F*_(2, 42)_ = 8.92, *p* = 0.001, η_*p*_^2^ = 0.30] was also significant. Simple effect analyses showed that the frontal LPP amplitude in the pre-test was higher than that in the post-test (*p* < 0.001), and the central–parietal LPP amplitude in the pre-test was higher than that in the post-test (*p* = 0.001), whereas the parietal–occipital LPP showed no significant difference in amplitude between pre-test and post-test (*p* = 0.64) (see Table 3). These results are consistent with hypothesis 3. The frontal and central–parietal LPP amplitudes decreased after repeated watching, whereas the parietal–occipital LPP amplitude was not obviously influenced by repetitions.

However, the semantic-focused group showed a strikingly different pattern, which was consistent with hypothesis 4. The main effects of valence [*F*_(2, 48)_ = 5.63, *p* = 0.006, η_*p*_^2^ = 0.19] and test [*F*_(1, 24)_ = 6.19, *p* = 0.02, η_*p*_^2^ = 0.21] were significant. Importantly, the Region × Test interaction was also significant [*F*_(2, 48)_ = 8.45, *p* = 0.001, η_*p*_^2^ = 0.26]. Further analyses showed that the frontal LPP amplitude in the pre-test was similar to that in the post-test (*p* = 0.64). However, the post-test central–parietal LPP amplitude was higher than the pre-test central-parietal LPP amplitude (*p* = 0.004). Similarly, the post-test parietal–occipital LPP amplitude was higher than the pre-test parietal–occipital LPP amplitude (*p* < 0.001) (Table 3). These results indicated that the central–parietal and parietal–occipital LPP amplitudes were enhanced after repeated watching, whereas the frontal LPP amplitudes were not influenced by repetition.

#### Standardized Low Resolution Tomography Analysis

**Figure 4:**
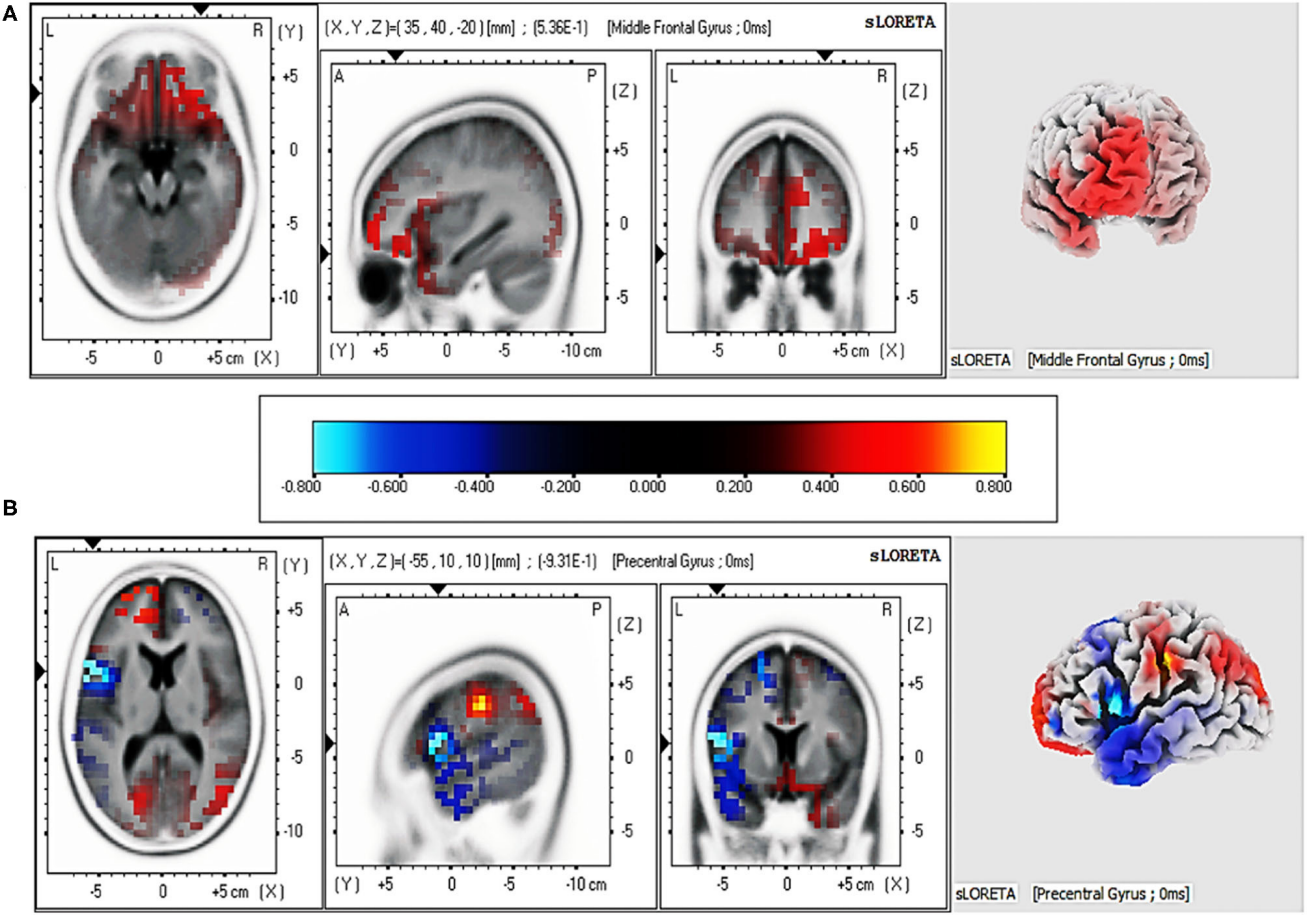
Regions with the most significant difference in current intensity between pre-test and post-test from sLORETA at 1,000–2,500 ms for two groups. **(A)** Brain regions activated by affective valence. **(B)** Brain regions activated by semantic valence.

We explored the source location related to the LPP (1,000–2,500 ms) for each group by sLORETA. To identify the cortical regions of the changes between pre-test and post-test in judging affective valence and semantic valence, the voxels with significant differences (*p* < 0.05) were compared in each group. The MNI coordinates, and Brodmann areas (BAs) of these regions and their corresponding structures are displayed in Table 4. In the comparison of the pre-test and post-test conditions, in the feeling-focused group, stronger activation was observed during the pre-test relative to the post-test in the middle frontal gyrus (x = 35, y = 40, z = −20; BA 11/10) and the superior frontal gyrus (x = 30, y = 45, z = −15; BA 11). In the semantic-focused group, the precentral gyrus (x = −55, y = 10, z = 10; BA 44) showed significantly reduced activation during the pre-test than during the post-test. Figure 4 displayed the cortical areas with the most significant difference elicited by the pre-test relative to the post-test in the two groups. These results indicated that the changes in the pre-test and post-test brain areas activated by the two groups were different when the participants judged the different modes of valence.

**Table 4:** Brain regions which showed significant differences in activation between pre-tests and post-tests at the late LPP latency for affective and semantic valence.

**Valence type**	**Structure**	**Lobe**	**Brodmann area**	**MNI coordinates (X, Y, Z)**
Affective valence	Middle frontal gyrus	Frontal	11	(35, 40, −20)
	Middle frontal gyrus	Frontal	11	(30, 40, −20)
	Middle frontal gyrus	Frontal	10	(35, 60, −5)
	Middle frontal gyrus	Frontal	10	(35, 60, 0)
	Superior frontal gyrus	Frontal	11	(30, 45, −15)
Semantic valence	Precentral gyrus	Frontal	44	(−55, 10, 10)
	Precentral gyrus	Frontal	44	(−60, 15, 10)
	Precentral gyrus	Frontal	44	(−60, 10, 15)
	Precentral gyrus	Frontal	44	(−50, 10, 10)
	Precentral gyrus	Frontal	44	(−55, 10, 15)

## Discussion

The primary purpose of this study was to demonstrate differences between the two modes of valence by comparing their spatiotemporal dynamics. Behaviorally, (1) the valence intensity of the feeling-focused group decreased in the post-test compared with that in the pre-test, while the semantic-focused group did not show differences between the two tests. At the neural level, (2) both groups showed significantly decreased frontal–central N2 amplitude and larger early LPP amplitudes in central–parietal and parietal–occipital areas in the post-test. Most importantly, (3) the feeling-focused group elicited larger frontal and central–parietal LPP amplitudes and stronger middle frontal gyrus activation during the pre-test than during the post-test. In contrast, the semantic-focused group elicited larger central–parietal and parietal–occipital LPP amplitudes during the post-test and stronger precentral gyrus activation during the pre-test. All these data suggested that although the early processes of affective and semantic modes of valence are similar, the late neural activation and cognition processes are different.

Specifically, after repeated viewing, the changes in valence intensity were different for affective and semantic valence. Current behavioral data replicated the results of a prior study (Itkes et al., 2017), suggesting that affective valence was sensitive to habituation, while semantic valence was not. These findings reconfirmed that the dissociation of affective and semantic valence could be achieved through repeated exposure in the habituation paradigm. Specifically, after repeated viewing, the self-reported intensity of affective valence decreased, whereas the intensity of semantic valence did not.

The two groups showed the attenuation of N2 amplitudes during the period of 210–280 ms. N2 amplitude attenuation has been found in early time windows in repetition studies (Ferrari et al., 2010; Bradley et al., 2015). This N2 inhibition effect is generally seen as increased perceptual fluency; that is, the recently presented stimuli can facilitate the later perceptual process (Codispoti et al., 2007; Ferrari et al., 2010). In distributed repetition, images are repeated many times at short intervals, which may establish short-term memory representations and, thus, facilitate the later perception of stimuli (Ferrari et al., 2015). Therefore, although the participants in this study followed different instructions, they all viewed the same pictures many times, and their perception of the pictures in the early stage was faster and smoother. In other words, whether the subjects judged affective or semantic valence, repeated viewing promoted their perceptual fluency.

According to the results, the changes in the early LPPs in the central-parietal and parietal-occipital sites were the same for both affective and semantic valence, with the amplitudes in the post-test being more positive than those in the pre-test. These findings are similar to the old–new effect in time course and scalp distribution of the recognition studies in which the old items in the recognition task elicit more positive amplitudes in posterior areas compared with the new items (first presentation) (Curran and Doyle, 2011; Godbole et al., 2014). Recognition is considered to be the re-retrieval of stimulus traces, and multiple retrievals can result in the enhancement effect of posterior LPP amplitudes (Weymar et al., 2013a). Distributed repetition is similar to a spaced process of repeated recognition, which increases the possibility of episodic retrieval. Previous distributed repetition studies for words or pictures have also found enhanced LPP amplitudes over the central–parietal region in the time window of 500–800 ms (Finnigan et al., 2002; Nelson et al., 2013). Further research has indicated that repetitions facilitate retrieval processes even in the absence of an explicit recognition task (Ferrari et al., 2013). Therefore, this retrieval process may be spontaneous and independent of the subsequent task. The findings add to the results of these studies, and enhancement effects of early LPP amplitudes can be found for both groups. The results suggested that whether the participants were asked to judge affective or semantic valence, they would spontaneously realize after repeated viewing that the pictures were old stimuli that were previously presented. Accordingly, this retrieval process of an old stimulus could elicit higher amplitudes in the early LPP amplitudes for both groups, regardless of the kinds of valence that the participants judged.

The key findings of this study were the differences in the changes in amplitudes, scalp distribution, and source localization of the LPP for the two groups during 1,000–2,500 ms, which may reflect the different neural mechanisms when judging affective valence or semantic valence. In the feeling-focused group, the LPP amplitudes in the frontal and central-parietal regions were weaker in the post-test than in the pre-test. The results are in accordance with the study of Codispoti et al. (2006). In their research, participants viewed the pictures passively many times. Peripheral physiological indicators (e.g., heart rate and skin conductance) decreased rapidly in the initial stage of repetition, and the amplitudes of LPP attenuated gradually. They explained the decrement as a reduction in the allocation of attention to stimuli caused by repeated viewing. Although the researchers did not ask the participants to feel emotional changes, further research has shown that reduced attention resources allocated to stimuli lead to a decline in emotional responses (Pessoa et al., 2002). However, the participants in this study were explicitly asked to judge affective valence, that is, to feel emotional changes during repeated viewings. We speculate that in this process, the attention of the participants that was allocated to the pictures gradually decreased because of repetitions, thus reducing the intensity of the emotional experience and resulting in a decrease in LPP amplitudes. Several studies have also shown that peripheral physiological indicators related to emotional responses, such as skin electricity and heart rate, show a trend of rapid habituation with increasing repetitions (Klorman, 1974; Bradley et al., 1993). Notably, the sLORETA results showed that the activation of the middle frontal gyrus in the pre-test was stronger than that in the post-test. According to previous studies, brain activities in the frontal cortex, such as the middle frontal gyrus and anterior cingulate gyrus, are linked to emotional responses (Davidson, 2000; Blair et al., 2007; Ertl et al., 2013). The result of this source location provided stronger evidence for the speculation. Therefore, when the participants judged the affective valence of pictures for the first time (first viewing) in this study, they might have experienced intense feelings. However, as the number of views increased, the intensity of the affective valence-related feelings decreased.

According to the instructions of the semantic-focused group, the participants judged the semantic valence of pictures based on their stored knowledge. The results showed that the post-test LPP amplitudes during the 1,000–2,500 ms gradually increased in central–parietal and parietal–occipital areas and were more positive than the pre-test amplitudes. Also, the results of sLORETA indicated that precentral gyrus activation in the post-test was stronger than that in the pre-test. Previous studies have shown that activation of the precentral gyrus is often implicated in brain areas activated by semantic processing tasks (Seghier et al., 2004; Pexman et al., 2007). Moreover, studies have shown that deeper semantic processing could increase the activation of relevant brain regions and improve the accuracy of subsequent memory tasks (Otten et al., 2001; Fliessbach et al., 2010). In the repetitive process of judging semantic valence, the participants repeatedly extracted and imagined the background stories of the details in pictures, by which they may have deeply processed the pictures with semantic elaboration (Kaneda et al., 2017). Therefore, the enhancement of LPP amplitudes might be interpreted as evidence that the participants continuously retrieve information from their stored knowledge (semantic elaboration). In other words, when viewing a picture for the first time, participants may simply process the contents of it, while the last time they view the picture, they could imagine a complete back story and then judge the semantic valence of the picture from their own experience.

The spatiotemporal dynamics of affective valence and semantic valence are different, and the differences are reflected not only in the changes in LPP waveforms but also in the activation of brain structures. The dissociation between these different psychological processes suggests that affective and semantic are two different modes of the valence system. Affective valence focuses on the process of experiencing emotion, whereas semantic valence represents semantic extraction. The results have important implications for emotion-related ERP studies. Researchers need to specify whether the purpose of a study is to measure the valence of emotional responses or the valence of stored semantic knowledge. These two modes of valence have differences in ERP waveforms and activated neural structures, which may confound the results and make them challenging to explain.

This study still has some limitations that offer directions for future research. First, the ERP technology has difficulties in locating accurate brain neural circuitries with a low spatial resolution. sLORETA is an insufficient method to use to fully identify the neural activities involved in the affective and semantic modes of valence. Previous fMRI studies have shown that emotional perception or semantic retrieval induced by pictures can activate multiple different brain regions, such as the left ventrolateral prefrontal cortex, amygdala, and anterior cingulate (Matsuda et al., 2013; Sabatinelli et al., 2013). Therefore, future studies should use precise spatial positioning techniques, such as fMRI, to reflect the brain regions activated by the two modes of valence, so that the evidence for neural dissociation will be stronger. Second, we confined the sample to female participants. Although this methodological approach effectively controls for sex differences in affective habituation (Andreano et al., 2014), it limits the generalizability of the findings. Future studies could replicate the present findings among male samples or among samples with balanced sex distribution. Third, to rule out the fatigue effect, it is better to show a new set of images to participants after repeated viewing. However, adding a new set of pictures for the participants to rate would increase the time of the EEG study, which would increase the fatigue of the participants instead. This is because the subjects needed to wear electrode caps and sit in a quiet experimental room. In addition, Itkes et al. (2017) adopted a similar design, and general fatigue did not affect the results. Even so, future studies need to be more tightly controlled.

Overall, the results describe the spatiotemporal dynamics underlying the observed behavioral changes for the affective and semantic modes of valence, which may imply different psychological processes. Thus, these findings provide electrophysiological evidence for the diversity of the valence system for the first time. Individuals may have two distinct patterns for processing affective valence and semantic valence. Future valence-related studies need to use clear instructions and avoid confusion between affective and semantic valence.

## Data Availability Statement

The raw data supporting the conclusions of this article will be made available by the authors, without undue reservation.

## Ethics Statement

The study conformed to the principles of the Declaration of Helsinki (World Medical Association, 2013) and was approved by the Academic Committee of the Ministry of Education of Key Laboratory of Modern Teaching Technology, Shaanxi Normal University in China. All participants provided informed consent after the procedures were fully explained, and were paid for their participation in the study. The patients/participants provided their written informed consent to participate in this study.

## Author Contributions

LW, XL, and SQ conceived and designed the study, conducted the interpretation of data and wrote the first draft of the manuscript. SQ, LW, and SX performed the experiments and statistical analyses. ZP and XY critically revised the manuscript for important content and provided general advice. All the authors have approved the final manuscript.

## Conflict of Interest

The authors declare that the research was conducted in the absence of any commercial or financial relationships that could be construed as a potential conflict of interest.

## Footnotes

^1^The ID numbers of the IAPS pictures used were the following: positive (1463, 1710, 2070, 2071,5831); negative (2141, 2661, 2681, 3220, 3230, 6021, 7359, 9185, 9250, 9421, 9600, 9622); neutral (1600, 2411, 2516, 2593, 2749, 7004, 7081, 7175, 7211, 7493, 7500, 7547).
